# Computational prediction of human disease-related microRNAs by path-based random walk

**DOI:** 10.18632/oncotarget.17226

**Published:** 2017-04-19

**Authors:** Israel Mugunga, Ying Ju, Xiangrong Liu, Xiaoyang Huang

**Affiliations:** ^1^ Department of Computer Science, Xiamen University, Xiamen, 361005, China

**Keywords:** systems biology, microRNA, disease-related microRNA prediction, path-based random walk

## Abstract

MicroRNAs (miRNAs) are a class of small, endogenous RNAs that are 21–25 nucleotides in length. In animals and plants, miRNAs target specific genes for degradation or translation repression. Discovering disease-related miRNA is fundamental for understanding the pathogenesis of diseases. The association between miRNA and a disease is mainly determined via biological investigation, which is complicated by increased biological information due to big data from different databases. Researchers have utilized different computational methods to harmonize experimental approaches to discover miRNA that articulates restrictively in specific environmental situations. In this work, we present a prediction model that is based on the theory of path features and random walk to obtain a relevancy score of miRNA-related disease. In this model, highly ranked scores are potential miRNA-disease associations. Features were extracted from positive and negative samples of miRNA-disease association. Then, we compared our method with other presented models using the five-fold cross-validation method, which obtained an area under the receiver operating characteristic curve of 88.6%. This indicated that our method has a better performance compared to previous methods and will help future biological investigations.

## INTRODUCTION

MicroRNAs (miRNAs) are a class of small, endogenous RNAs that are 21–25 nucleotides long. MiRNAs are found in plants, animals, and some viruses, and function in RNA silencing and post-transcriptional regulation of gene expression [1, 2]. MiRNAs are involved in many diverse biological processes, such as development, differentiation, apoptosis, and viral infection [3]. Increasing evidence implicates miRNAs in human disease development, progression, prognosis, diagnosis, and evaluation of treatment response [4–6]. Since the first miRNA (lin-4) was discovered from C. *elegans* 20 years ago, many miRNAs have been annotated in various species with experimental and computational methods [7]. However, the discovery of disease-related miRNA via existing biological experimental methods is expensive and time-consuming [8]. Thus, computational prediction methods are significant techniques for identifying the most promising miRNA-disease associations prior to additional experimental examinations. Many databases have been developed to accumulate miRNA data. The Human MiRNA Disease Database (HMDD) [9] is an online database that provides complete information on miRNA deregulation in various human diseases. MiRbase [10] is a comprehensive miRNA database that contains the sequences of precursor miRNAs, mature miRNAs, miRNA hairpin structure, and miRNA targets. OncomiRDB is a manually curated miRNA-cancer association database that contains more than 300 miRNAs and 2259 miRNA-cancer associations [11]. dbDEMC [12] is an integrated database that is designed to store and display differentially expressed miRNAs in human cancers. MiR2disease [13] is a manually curated database that provides a comprehensive resource from miRNA deregulation in various human diseases. The identification of miRNAs that underlie human diseases is an important goal of biomedical researchers. However, one major issue in miRNA studies is the lack of enough bioinformatics methods that predict potential miRNA-disease associations.

Computational methods for predicting miRNA and related diseases have been proposed to overcome this major issue. Computational predictions indicate that miRNAs, which account for at least 1% of human protein-coding genes, regulate protein production for thousands of human genes. Most computational predictive methods for miRNA-disease associations are based on the hypothesis that miRNAs with similar functions tend to associate with a common disease and that diseases with shared similar phenotypes likely share common miRNA [14–16]. Therefore, different methods have been proposed to predict miRNA and disease association such as random walk-based methods. In 2008, Xu et al. [17] proposed miRank, a ranking algorithm based on random walk. They tested their method on *Homo sapiens* genomes and achieved a good accuracy. In 2012, Chen et al. [18] adopted a global network similarity measure and developed Random Walk with Restart for miRNA-disease Association (RWRMDA) to infer potential miRNA-disease interactions by implementing random walk on a miRNA–miRNA functional similarity network. Prediction of disease-related miRNA has proposed in various ways. A robust regularization path for *v*-support vector classification based on lower upper decomposition with pivoting is a method present in [19]. This method uses a regularization parameter *v* as the assistance of adjusting the number of support vectors and margin errors.

In 2014, Liu et al. [20] developed a random walk with restart method. Using this model, they first revealed the limitations of previous computational methods, and then implemented random walk with restart on a heterogeneous network to infer potential miRNA-disease associations. To declare these limitations, the first limitation has stated as the use of single dataset, second limitation as an inadequacy of disease semantic similarity and the third limitation as an overestimation of the predictive accuracy. They applied their method to diseases with no known related miRNAs. The majority of the top 30 candidates were confirmed by various databases.

In addition, many researchers have proposed different methods of predicting miRNA-related diseases. In 2009, Jiang et al. [21] proposed the miRNA-disease association prediction technique, wherein miRNA–miRNA and human phenome–miRNA functional similarity networks were constructed. The score for all miRNAs was computed using cumulative hypergeometric distribution [21]. Chen et al. [22] anticipated HGIMDA method as a graph inference by integrating miRNAs similarity, diseases similarity and Gaussian interaction profile kernel similarity into a heterogeneous graph. To analyze, predict, and providing possible lncRNA-disease associations is another problem stated by Chen et al. [23]. Improved random walk with restart to predict lncRNA with related disease method has been proposed to improve the traditional limitations of random walk with restart method. It also based on an integration of data similarities such as diseases and lncRNA, and applying a random walk to predict novel lncRNA-disease.

In 2015, Xuan et al. [24] proposed MIDP, a predictive model for disease-related miRNA. In this method, the prediction process is modeled as random walk on a miRNA network that is derived from miRNA-associated diseases. For a specific disease with some related miRNAs, a random walker starts at a known related miRNA node with equal probability. An extension method, MIDPE, was specifically proposed for diseases without any known related miRNAs. This model obtained a higher predicted accuracy [24]. In addition to random walk-based methods, many other methods have been developed to support biological examinations.

Xuan et al. [25] presented HDMP, a method that is based on weighed *k* and most similar neighbors, for miRNA-disease association prediction. The functional similarity of their method combined the similarity of information content of disease terms and phenotype similarity between diseases. Based on the hypothesis that miRNAs with similar functions tend to associate with a common disease and that diseases with shared similar phenotypes likely share common miRNA [14–16], Chen et al. [26] developed WBSMDA to predict relationship among miRNAs and diseases. By combining data from disease-related miRNAs, similarity miRNAs, disease similarity and similarity of Gaussian interaction profile kernel, a score has been calculated to predict new miRNA-disease associations. The model evaluation has been conducted based on Leave-one-out cross validation method to verify its performance. However, this method attained a better performance, it has a limitation; such that it was developed based on the hypothesis in [14–16], which may cause unfairness to miRNAs which have a lot number of related diseases.

Chen et al. [27] used three network-based similarities (miRNA-based similarity inference, phenotype-based similarity inference and network-consistency-based inference) to infer potential miRNA-disease associations. Another model proposed based on matrix completion algorithm has been proposed to predict miRNA-disease associations based on the known diseases and miRNAs. Li et al. [28], proposed MCMDA model which based on updating the adjacency matrix feature for known related miRNAs and disease.

Zou et al. [29] presented Pretata, a predictive method to identify TATA-binding proteins. This model is an applied advanced machine learning methods which facilitate to identify TBP (TATA-binding proteins) and protein detection from primary sequences.

Le [30] constructed a miRNA function network based on common targets and applied four network-based methods to infer novel disease-related miRNAs. Mørk et al. [31] presented miRPD to infer miRNA-disease associations by coupling known and predicted miRNA–protein associations with protein–disease associations. Zeng et al. [32, 33] suggested two predictive methods on multipath to predict miRNA-disease associations based on heterogeneous network of diseases and miRNAs. To calculate the similarity between objects, HeteSim_Multipath (HSMP) method was proposed to combine scores form heterogeneous similarity paths with constant. The second method, HeteSim_SVM (HSSVM) uses machine learning method to combine heterogeneous similarity to measure scores without a constant.

Zou et al. [34] proposed KATZ and CATAPULT, which are based on social network analysis, to infer potential miRNA-disease associations. Gu et al. [35] developed a method based on the *v*-support vector regression was proposed to overcome the challenge that presented by the method of Schölkopf et al. [36]. The initial adjustments proposed in this work as an additional term to overcome this challenge where the parameter *v* has the role of monitoring the support vectors based on Karush–Kuhn–Tucker (KKT) conditions to make an initial solution for the incremental learning. Chen et al. [37] introduced a research review about lncRNA and disease associations. In this review, Chen et al., first introduced functions of lncRNA, five important of lncRNA-disease associations, precarious disease-related lncRNA and finally available important lncRNA-database related to sequence, expression, and function. Therefore, a state-of-the-art computational method has been proposed to identify disease and lncRNA.

Chen et al. [38] developed a machine-learning method, Regularized Least Square for MiRNA-disease Association (RLSMDA), to discover the potential relationships between diseases and miRNAs. Based on the hypothesis that lncRNAs with similar functions tend to be related with diseases that have similar functions, Chen et al. [39] developed FMLNCSIM, a model that computing a functionally similarity between lncRNA on a large scale by combining information content and fuzzy measure’ concept to the directed acyclic graphs disease. Zeng et al. [40] first presented three general problems in the prediction of miRNA-disease associations. The problems were styled as follows; the lack of similarity among miRNAs stated to be the first challenge in their research, and the second problem defined as inadequate miRNA-disease associations and lastly, the third problem distinct as not enough availability number of negative samples for investigating miRNA-disease relationship. Therefore, they proposed a matrix completion method which is based on integrating multiple feature sets to conquer the challenges [40].

Besides, another computational model to infer miRNA-disease association type pairs has been developed by Chen et al. [41]. RBMMMDA developed based on fact that all the previous computational techniques may only predict binary associations among miRNAs and diseases. Therefore, this method has been develop to conquer this challenge and can predict four different multiple types of miRNA-disease relationship.

Based on the hypothesis that functionally related microbes share the similar interaction and non-interaction pattern diseases, Chen et al [42] developed a model of KATZ measure for Human Microbe–Disease Association prediction (KATZHMDA). Therefore, this model was constructed based on a Gaussian interaction profile kernel similarity from disease and related microbe. To state this method thus, the similarity network derived from microbe-disease for Gaussian interaction profile kernel was contained by two main steps. Step one is that, every interaction profile of microbe indicated as binary vector which converts the existence or lack of the relationship among disease and microbe. In step two, based on interaction profiles, the Gaussian interaction profile kernel among a pair of microbe was computed. MiRNAs involve in various biological processes. Fungal infection is one of the infections that developed through miRNAs and predicting fungus-causing diseases became also attractive. Therefore, Chen et al. [43] proposed a computational model NLLSS, a semi-supervised learning technique used to combine different kind of information to predict potential synergistic drug combinations.

One the hand, we cannot ignore classification in this research; it helps in designing classifiers in real-world problems. By using two finite mixture models to capture the structural information from different classes, Gu et al. [44] presented a structural minimax probability machine (SMPM) to solve a sequence of SOCP (second order cone programming) problems through a binary search procedure. The method has further proposed a nonlinear SMPM model based on linear SMPM by using kernelization techniques. Predicting disease-related miRNA has been reported in different ways.

Although the proposed methods have successfully predicted miRNA-disease associations, these methods have some drawbacks. The most commonly reported drawback is the poor dataset quality of some methods, which causes poor performance. Some methods evaluate disease–miRNA similarity, miRNA similarity, and disease similarity by using disease data only, which possibly results cause bias that disregards miRNA features.

Therefore, we propose a computational prediction method for potential miRNA-disease associations. Our proposed model is based on path-based features and random walk. MiRNA functional similarity network [45], disease similarity network [46], and known miRNA-disease associations [9] were integrated in our work which ranked every miRNA of a given disease. High prediction ranks were predicted to have high probabilities as potential candidates of a given disease. Experimental results demonstrated that our method effectively predicted potential miRNA-related disease candidates. Known miRNA-disease association databases were applied to evaluate the performance of our proposed method.

## RESULTS

### Evaluation of prediction performance

**Figure 1:**
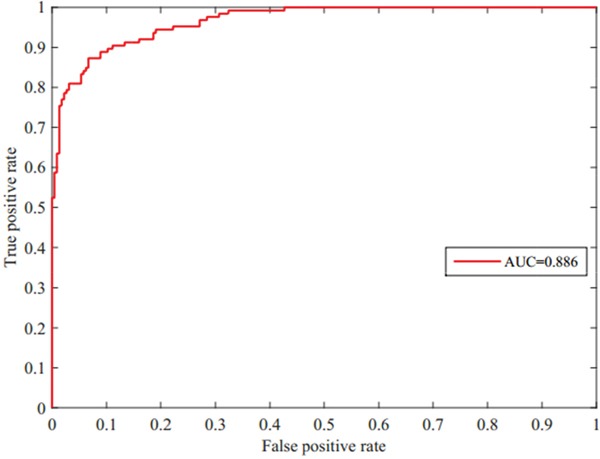
ROC curve and AUC=0.886 value of our predictive model for miRNA-disease associations by five-fold cross validation

We present a computational prediction method that is based on random walk and graph theory. We introduced the five-fold cross validation method to evaluate the strength of our method based on random walk and the construction of RDnet (miRNA and disease network). The datasets were randomly divided into *n* subsets, where *n–1* subsets were used for model construction to predict potential candidates. The remaining subset was used to test the performance of our model. A receiver operating characteristic (ROC) curve was applied to evaluate the strength of our proposed method. A ROC is designed by varying the achieved threshold. The numeric representation of ROC is the area under the curve (AUC). The ROC is shown in Figure 1. The horizontal axis in the plot represents false positive rate (1–specificity) and the vertical axis represents true positive rate (sensitivity). The true positive rate (TPR) refers to the percentage of true associations with scores that are higher than the given threshold. On the other hand, the false positive rate (FPR) is the ratio of the successfully predicted miRNA-disease associations to all known miRNA-disease associations. FPR refers to the percentage of associations with scores that are lower than the given threshold. Mathematical formulas of TPR and FPR are given below:

$$\text{TPR}=\frac{\text{TP}}{\text{TP}+\text{FN}}$$ (1)

$$\text{FPR}=\frac{FP}{\text{TN}+\text{FP}}$$ (2)

where true positive (TP) denotes the number of known associations with scores are higher than the given threshold. By contrast, false negative (FN) denotes the number of known associations with scores that are lower than the given threshold. True negative (TN) denotes the number of unknown associations with scores that are lower than the given threshold. False positive (FP) denotes the number of unknown associations with scores that are higher than the given threshold.

### Comparison with other prediction methods

Models by Xuan et al. [24] (MIDP and MIDPE, 2015), Chen and Yan [38] (RLSMDA, 2014), Chen and Zhang [27] (Chen's method, 2013), Shi et al [47] (Shi's method, 2013), Xuan et al. [25] (HDMP, 2013) and Chen et al. [18] (RWRMDA, 2012) comprise the majority of predictive computational methods that have been presented in previous years. MIDP, RWRMDA, and HDMP have exhibited considerably superior performances, but were developed based on the association data from earlier versions of HMDD [9]. Although we cannot ignore the performances of these methods, our proposed method is compared with MIDP as the only method that was implemented based on association data from the latest database [9] in 2013. MIDP [24] had a good predictable performance, extending to miRNA with no known related disease. However, the performances of these methods have not ignored. The comparison of our method with these methods has shown in Table 1 which confirms a higher predictive accuracy of our method compared with MIDP [24], RWRMDA [18], HDMP [25], and Chen's method [27].

**Table 1:** Prediction results of our method and other methods for 11 diseases with more than 100 related miRNAs in terms of accuracy (%) using five-fold cross validation

Diseases name	Our method	MIDP	RWRMDA	HDMP	Chen's method
Breast Neoplasms	**0.865**	0.854	0.785	0.801	0.653
Colorectal Neoplasms	**0.879**	0.845	0.793	0.802	0.662
Glioblastoma	**0.821**	0.786	0.68	0.70	0.607
Heart failure	**0.843**	0.821	0.722	0.77	0.761
Hepatocellular Carcinoma	**0.832**	0.807	0.749	0.759	0.613
Lung Neoplasms	**0.904**	0.876	0.876	0.835	0.606
Melanoma	**0.869**	0.837	0.784	0.79	0.642
Ovarian Neoplasms	**0.947**	0.923	0.882	0.884	0.644
Pancreatic Neoplasms	**0.968**	0.945	0.871	0.895	0.684
Prostatic Neoplasms	**0.898**	0.882	0.823	0.854	0.629
Stomach Neoplasms	**0.854**	0.821	0.779	0.787	0.628

From the above, we compared the accuracies of our method with MIDP [24], RWRMDA [18], HDMP [25], and Chen's method [27] for 11 diseases with more than 100 related miRNAs. The comparative analytical results of our method are presented in bold numbers.

In the analysis of all methods our method, MIDP [24], RWRMDA [18], HDMP [25], and Chen's method [27] held different parameters for tuning as follow: for our computational prediction method, the parameter **M** was set in the range of 0.1 to 0.9. Our method achieved a better performance when M=0.7. The highest scores from a given disease with miRNAs were confirmed as potential candidate miRNA-related diseases. The parameters **r_Q_** and **r_U_** of MIDP [24] were selected from (0.1,0.2,…,0.9) such that **r_Q_** was greater than **r_U_**. The parameter *r* of RWRMDA [18] ranged from 0.1 to 0.9. The parameter *k* of HDMP [25] diverged from 1 to 50. The parameter *r* for Chen's method [27] varied from 0.1 to 0.9. The summary of these parameters is shown in Table 2 below.

**Table 2:** Different parameters used in the prediction of miRNA-related disease

Methods	Our method	MIDP	RWRMDA	HDMP	Chen's method
**Range Parameter**	0.1-0.9 (**M**)	0.1-0.9(**r_Q_,r_U_**)	0.1-0.9 (**r**)	1-50 (**k**)	0.1-0.9 (**r**)

## DISCUSSION

We present a computational prediction method for ranking all unknown miRNAs that are related to a given disease. The high accuracy of our method has shown that supporting biological tests can reliably predict miRNA and related diseases. Comparing our method with other prediction models proved that its predictive capabilities. We first constructed a miRNA-disease network(RDnet) derived from [9]. Then, we built our model based on random walk and graph theory where the walker starts walking form a known miRNA-related disease with equal probability. From known associations, the walker's goal was to reach the neighbor of a targeted disease, which is the unknown association to calculate the similarity score as the relationship. The weights were set as similarity scores between unknown miRNA and a given disease. Thus, the highest scores were set as potential candidates of miRNA-disease associations, as proven by public databases. The most challenging problem is that the majority of miRNAs have no known related diseases. Moreover, many diseases also have a small amount of related miRNAs. This means that a high number of false negatives exists massively challenging this research field. The construction of consistent prediction models with better accuracies will help to overcome these challenges. Furthermore, using different features to discover the similarities between miRNA-disease associations will be useful in future studies.

## MATERIALS AND METHODS

We combined different data set to overcome the results of poor performances. The datasets that were utilized in this study were downloaded from the following databases as follows.

### Disease phenotype similarity data

We downloaded the disease phenotype similarity scores from the MimMiner which was developed by Van Driel et al. [46]. They computed a phenotype similarity score per phenotype pair via text-mining analysis of their phenotype descriptions in the Online Mendelian Inheritance in Man database [48]. The phenotypic similarity scores were successfully used to predict and prioritize disease-related protein-coding genes [49, 50].

### The human miRNA-disease association data

We downloaded experimentally verified miRNA-disease association in [9]. HMDDv2 is a database that contains experimentally supported data for miRNA-disease associations. These data are manually curated from publications. In our experiment, we used 578 miRNAs and 382 diseases from the 2013 released of HMDD, which had 10,381 entries [9].

### MiRNA–miRNA functional similarity data

The miRNA–miRNA functional similarity scores were downloaded from http://www.cuilab.cn/files/images/cuilab/misim.zip [45]. In this dataset, a functional similarity score per miRNA pair is calculated based on the observation that genes with similar function are often associated with similar diseases [14–16].

### Construction of RDnet (miRNA and disease network)

The key to network-based predictions of potentially disease-related miRNA is the calculation of similarity network among disease and miRNA over networks. The construction of miRNA and disease similarity network significantly affects the prediction of miRNA-related disease. The network was derived from [9]. The graph theory was used as a method for connecting different nodes and the walker to measure similarity nodes. Therefore, for all known disease-related miRNAs, if the miRNA *m_i_* (*i*=1, 2, 3… *n*) is related to any disease *d_i_,* this relationship between miRNA and disease was set to be 1, otherwise 0. This helps us to obtain the number of miRNAs that is connected to disease (*d*). Then, we extract all unknown miRNAs for the prediction. We ranked all unknown miRNAs. The higher-ranked miRNAs were confirmed as potential candidates of a given disease (*d*).

$$\left\{ \begin{array}{l} 1\text{ if }m_{i}=d_{i,}i=1,\;2,\;3...n\\ 0\;\;\;\;\;\;\;\text{otherwise}\; \end{array}\right.$$ (3)

From the dataset, the large numbers were unknown miRNA that caused greater challenges for researchers in the prediction and discovery of new miRNA-disease associations. Moreover, several diseases have no related miRNA. Therefore, random walk and path theory were combined to overcome these challenges.

### Random walk and graph theory

A random walk consists of a sequence of vertices, which are generated from a start vertex by first selecting an edge, then traversing the edge to a new vertex, and finally repeating the process. We constructed a strongly connected graph from RDnet network. Then the fraction of time the walker spends at various vertices of the graph converges to a stationary probability distribution. After constructing RDnet, we solved our problem using random walk to calculate the miRNA score for a given disease. Then, high-ranked miRNAs were stated as potential candidates for miRNA-related disease. Initial states of the walker are highly dependent on the start state of the walk. Therefore, the walker starts walking from a known disease node until it reaches to related miRNA nodes with equal probability for each miRNA. Nevertheless, the probability that the walker arrives at each miRNA node is equal, the walker prefers the neighboring miRNA nodes in the network. Then, from the disease-related miRNA node, the walker will continue walking to unknown miRNAs of a given disease. The probability that unknown miRNA relates with a given disease is considered by the similarity score between unknown miRNA with a given disease.

Two nodes are related if the weight of an edge between two vertices is higher than a given threshold. However, in random walk, the next step does not depend on the previous history of steps, only the current position/node of the moving walker. This is the case for random walk on a directed graph (randomly selecting an outgoing edge out of *d*… disease to leave from) and walks on a weighted graph (select an edge with probability proportional to its weight). In our method, we modeled the score as a weight of our graph after calculating the similarity score. Here, we have our network as the undirected graph.

### Path-based random walk

We design our experiment based on random walk and graph theory. For a given disease *d*, a known miRNA, miRNA-related disease candidates, and their relationship were modeled as a weighted graph G. Given a weighted graph G = (V, E), where *v* ∈ *V* in G represents the vertices of a known miRNA-related disease, miRNA–miRNA similarity, disease–disease similarity, and miRNA-disease potential candidates, *e ∈ E* ⊆ *V × V* captures the relationship between two vertices that are linked by the edge. Moreover, the weight *w* of an edge *e* measures the relationship between a given disease and unknown miRNAs after calculating the similarity score. The higher the *w*, value is, the better probability that two vertices are associated with a group of similar diseases. We aim to calculate the ranking order by measuring the similarity score of all unknown nodes to obtain the potential miRNA candidates of a given disease. From a given disease-related miRNA nodes, the walker starts walking to their neighboring nodes with equal probability. The proposed method is illustrated below.

**Figure 2:**
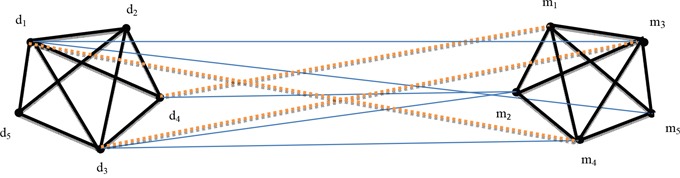
Illustration of the proposed method based on random walk and graph theory derived from RDnet

MiRNAs that possess similar functions are implicated in similar diseases and vice versa [14–16]. The graph was labeled as undirected; from the Figure 2, disease network and miRNAs network were combined to form a miRNA-related disease network that is derived from RDnet (miRNA and disease network). Blue straight lines from diseases to miRNAs signify that disease nodes and miRNA nodes are related. The walker starts walking from known related miRNA-disease with equal probability. The probability that the walker starts from diseases (*d*_1_, *d_2_, d_3_*…*d*_n_) to miRNAs (*m*_1_, *m_2_, m_3_*…*m*_k_) is equal to every known disease-miRNA node. Therefore, the walker proceeds to the closest neighbor of a related miRNA; the closer it is to the node, the higher it is likely to be connected. From Figure 2, *m_5_* to *m_1_*, and *m_5_* to *m_3_* are clearly shown as neighbors in the miRNA network. In addition, disease's network and miRNA's network have been constructed based on their functional similarity. Then, from RDnet, *d_1_* is strongly related to *m_5_*, *d_3_* is associated with *m_2_* and *d_1_* is also associated with *m_3_*. From the assumption that miRNAs share the same functional similarity and are related to similar diseases and vice versa [14–16]. Thus, the walker moves from *m_5_* and walks to its neighbor *m_4_*. Path-based random walk states that the walker follows the walking path from miRNA-related disease until it reaches to its neighbor. Therefore, the similarity score between a given diseases with any unknown miRNAs was calculated through known disease-associated miRNAs and disease network. The similarity score confirmed that *d_1_* and *m_4_* are likely to be associated as shown by the brown dotted lines in Figure 2. Graph theory and random walk were used interchangeably as the walker has to reach each node of the graph until converging; *d_4_* is associated with *m_2_* and *m_2_* has *m_1_* as its directly connected vertex neighbor. This means that when the walker reaches to *m_2_*, the probability of moving to its neighbors is equal to each unknown miRNA node; then the walker will stay in the unknown node to measure the similarity score. As the walker starts to walk from known nodes *d_3_*–*m_2_*, the walker will continue to the neighbor vertex *m_3_* to predict an association between vertices *d_3_* and *m_3_* as shown by the brown dotted line from nodes *d_3_* to *m_3_* in the Figure 2. Here, *m* stands for miRNA and *d* stands for disease. We thus calculated the similarity score from unknown miRNAs with a given query disease. The higher the similarity scores, the better the prediction that miRNA is a potential candidate of a given disease. A comparison of the top 10 miRNAs potential candidates are described in Table 3 as discussed above in section two (Results).

**Table 3:** The top 10 highest scores miRNAs potential candidates related to Hepatocellular carcinoma as confirmed by public databases

MiRNAs	Confirmation	Ranks
hsa-mir-507	dbDEMC2.0	1
hsa-mir-30e	dbDEMC2.0	2
hsa-mir-9-2	dbDEMC2.0;Mir2desease	3
hsa-mir-520f	dbDEMC2.0	4
hsa-mir-132	dbDEMC2.0	5
hsa-mir-424	dbDEMC2.0	6
hsa-mir-431	dbDEMC2.0	7
hsa-mir-34b	dbDEMC2.0	8
hsa-mir-149	dbDEMC2.0	9
hsa-mir-185	dbDEMC2.0	10

### Predictive algorithm for miRNA-related disease similarity score of potential candidates

**Inputs**: RDnet, denoted as *G (V, E, W)*; specific disease *d*.

**Outputs**: Top ranked *d*-related miRNA candidates.

1. Obtain diseases and miRNAs associations to form RDnet.
2. For *i*^th^ element of the initial probability *d(0)*, *d_i_(0)* (*1* ≤*i* ≤*N*, *N* is the number of vertices in the graph G);
3. If the *i*^th^ vertex, *d_i_* – *m_j_* is a known disease-related miRNA node (where *d_i_* is any disease node and *m_j_* is any miRNA node);
4. *d_i_ – m_j_* =1
5. else
6. *d_i_ –m_j_* =0
7. End If
8. End For
9. Initialize the walker vector *X=d(0)*
10. For each vertex *d_i_* (1≤*i*≤*N, d_i_* is the number of disease vertices in the graph G).
11. For each vertex *m_j_* (1≤*j*≤*N, m_j_* is the number of miRNA vertices in the graph G).
12. The walker is walking from *d_i_* – *m_j_* nodes until reaches to unknown neighboring miRNA nodes of disease-related miRNA node.
13. Calculate the sum of the probability that the walker arrives at each unknown miRNA node.
14. The steady-state that the walker will stay at the *m_j_* unknown miRNA node is used as the relevancy score between unknown miRNAs and a disease node.
15. End For
16. End For
17. All the unknown miRNAs nodes are ranked by their scores.
18. The unknown miRNAs with higher ranks are confirmed as potential candidates of *d*-related miRNA.
